# ﻿Phylogenetic analysis reveals a new net-winged beetle genus of Eurrhacini (Coleoptera, Lycidae) from the Pacific slopes of Central America and Ecuador

**DOI:** 10.3897/zookeys.1204.114932

**Published:** 2024-06-06

**Authors:** Elynton Alves Nascimento, Milada Bocakova

**Affiliations:** 1 Departamento de Engenharia Ambiental, Universidade Estadual do Centro-Oeste, Rua Professora Maria Roza Zanon de Almeida, s/n, Engenheiro Gutierrez, Irati–PR , CEP 84505-677, Brazil Universidade Estadual do Centro-Oeste Irati Brazil; 2 Department of Biology, Faculty of Education, Palacky University, Purkrabska 2, CZ-77140 Olomouc, Czech Republic Palacky University Olomouc Czech Republic

**Keywords:** Lycinae, Neotropical Region, new genus, new species

## Abstract

The first phylogenetic inference of Calopterini and Eurrhacini focused on *Calocladon* and related taxa was carried out. A data matrix composed of 46 species and 51 morphological characters was assembled and analyzed using parsimony and model-based approaches. Eurrhacini were recovered monophyletic. Furthermore, phylogenetic analyses highly supported the *Calocladon* clade including also *Atlanticolycus*, *Cladocalon*, and *Gorhamium***gen. nov.** as its sister clade. Our trees consistently recovered monophyly of the new genus with two new species: *Gorhamiumbidentatum***sp. nov.** (Panama, Baru Volcano) and *G.unidentatum***sp. nov.** from the Pacific slopes of Ecuador. A revised key to the genera of Eurrhacini is given and illustrations of distinguishing characters are provided. Phylogenetic relationships of Eurrhacini and character evolution are discussed.

## ﻿Introduction

The Eurrhacini is a Neotropical lineage of Lycidae, which until recently was part of the tribe Calopterini in the broader sense (Bocakova 2003, 2005). However, the inclusion of Eurrhacini in Calopterini was challenged by the first molecular analysis of Lycidae (Bocak et al. 2008) which showed that *Eurrhacus* Waterhouse, 1879 is sister to the Oriental *Conderis*. Consequently, Eurrhacini was excluded from Calopterini (Bocak and Bocakova 2008) and elevated to the tribal rank. Nevertheless, inferring the Eurrhacini sister group is convoluted because DNA analyses proposed several candidates. Recent molecular trees on large data sets (Masek et al. 2018) recovered the Eurrhacini sister is either American Thonalmini or Oriental Lycoprogenthini, thus indicating that Calopterini and Eurrhacini are not sister lineages.

The placement of Eurrhacini in the Calopterini was based on their resemblance, as the two groups often have similar coloration. Eurrhacini, however, are characterized by a very long male terminal sternum, which is twice as long as that of the Calopterini, and a distorted phallus and phallobase. When established (Bocakova 2005), Eurrhacini included six genera. Of these, *Calocladon* Gorham, 1881 has a markedly elongated pronotum, *Lycoplateros* Pic, 1922 is characteristic by a conspicuous protuberance on the posterior margin of the pronotum, and *Haplobothris* Bourgeois, 1879 is easily distinguishable by the absence of secondary elytral costae. The remaining three genera (*Eurrhacus*, *Emplectus* Erichson, 1847, and *Neolinoptes* Nascimento & Bocakova, 2017) are less distinctive externally, but easily separated by the shape of the male genitalia.

Likewise, species of the recently discovered *Cladocalon* Nascimento & Bocakova, 2022, *Currhaeus* Nascimento, Bressan & Bocakova, 2020, and *Atlanticolycus* Nascimento & Bocakova, 2023 were originally placed in *Calocladon*, as they are similar to *Calocladon* and *Emplectus* (Nascimento et al. 2020; Nascimento and Bocakova 2022). However, showing great male genitalia disparity, they were assigned to generic rank. Recently, an examination of H. S. Gorham’s types from Panama and further research on material from Ecuador have revealed another previously hidden generic lineage described below. Here we elucidate phylogenetic relationships of the group and its placement within Eurrhacini by analyzing morphological data.

## ﻿Materials and methods

The morphological matrix is based on that of Bocakova (2005), updated by Nascimento et al. (2020) and Ferreira et al. (2023). The dataset was expanded by the inclusion of two recently proposed Eurrhacini genera (*Atlanticolycus*, *Cladocalon*) and the new one described here (altogether six newly coded species). Our final matrix (Table 1) is composed of 46 species and 51 characters (Suppl. material 1), including five outgroup taxa. Of these, ten characters were coded to multistates, 41 characters as binary. Eight additional characters (#44–51) were newly defined, other characters required the inclusion of new character states, or minor redefinition. Unknown and inapplicable characters were coded by a question mark “?”, or a dash “-”, respectively.

**Table 1.** Data matrix of 51 morphology-based characters of Calopterini and Eurrhacini used in phylogenetic analyses.

*Dictyopteraaurora*	011000000000000000000001000000000000000000-00-0-000
*Lygistopterussanguineus*	10-01-00000000000000001100000000-000000010-00-0-000
*Platerosbrasiliensis*	10-100000000001-0-00001100000000-001000000-00-0-0-0
*Conderissignicollis*	011000000000000000000000000010000000000001000-0-000
*Lycuspalliatus*	10-11-00000000000000000000000100-100000000-00-0-000
*Haplobothrisbasipennis*	11101-000100000110??????100000??0101000010-00-0-000
*Haplobothrisscapularis*	11101-000100000110101000100000000100000010-00-0-000
*Calocladontestaceum*	111100010100200111101100110000000021000001001110000
*Calocladonoculatum*	111100010100200111101100110000000021000001001110000
*Calocladonephippium*	111100010100200111101100110000000021000001001110000
*Atlanticolycuscamposgerais*	111?000101042001?11031??110000000000000001011112000
*Atlanticolycusjapi*	111?000101042001?11031??110000000000000001011112000
*Cladocalonchiriquense*	111?000101031100111011??100000000000000001001012011
*Cladocalonhistrionicum*	111?000101031100111011??100000000000000001001?12011
*Gorhamiumbidentatum*	1110000101032000211011??100002000000000001001011102
*Gorhamiumunidentatum*	111?00010103200121??????110002000000000001001111102
*Emplectusbimaculatus*	1110000???????????100111?0????00000??0?00100???????
*Emplectusapicalis*	11100001010020001-100111100000000000000001000---0--
*Currhaeusstriatus*	111?0001010-001-0-??????100000??1002000001000-0-000
*Currhaeuschampioni*	111?0001010-001-0-??????100000??1002000001000-0-000
*Currhaeusparanaensis*	111?0001010-001-0-??????100000??1002000001000-0-000
*Eurrhacustristis*	11110001210-201-0-??????100000??0001100001000---0--
*Eurrhacuspectinicornis*	11110001210-201-0-100111100000000001100001000---0--
*Eurrhacuskaboureki*	111?0001210-201-0-??????100000??0001100001000---0--
*Neolinoptesimbrex*	111100002100100011100101100000001001000001000-0-000
*Neolinoptesrubidus*	111100000100200011??????100000??1001000001000-0-000
*Lycoplaterosmimicus*	10-1000???????????100100?0????00-00?011001100-0-000
*Lycoplaterosdiversipes*	10-10001010020001110010000000000-001011001100-0-000
*Cyrtopteronmuhlenbecki*	11011-001000010110100000000000101000000001100-0-000
*Mesopteronriveti*	10-11-001000010110??????001000??-000000011100-0-000
*Falsocaeniameridanum*	11011-101000010110112000000000101000000011100-0-000
*Lycinellaopaca*	10-?01001001000100??????010000??-000000020-00-0-000
*Lycinellaparvula*	10-?01001001000100??????010000??-000000020-00-0-000
*Ceratopriomorphushumeralis*	111?01000001010110??????010100???000000011100-0-000
*Ceratopriomorphuspiceus*	111?01000001010110??????010100???000000011100-0-000
*Acroleptuschevrolati*	111?01010001010110??????010000??0000000010-00-0-000
*Metapteronsuturalis*	11111-000012010110111000000000000000000001000-0-000
*Calopteronapicale*	10-11-00001001011010200000000001-100000001000-0-000
*Cartagonumbernardi*	10-11-100010010110??????000000??-000000021000-0-000
*Leptoceletesbasalis*	10-11-01001001011010200000000000-010000001000-0-000
*Caeniadimidiata*	10-10001101000011010200000000000-010000001000-0-000
*Idiopteronflavocinctum*	10-11-11101001001010200000000000-010000021100-0-000
*Xenomorphonbaranowskii*	10-?---01000010110??????010000??--00000121100-0-000
*Lycomorphonelongaticolle*	10-?1-101000010110??????010000??-000000001000-0-000
*Lycomorphonbolivianum*	110?1-101000010110??????010000??1000000001000-0-000
*Lycomorphonangusticolle*	10-?1-101000010110??????010000??-000000001000-0-000

Phylogenetic analyses were conducted using maximum parsimony (MP), Bayesian (BA), and maximum likelihood (ML) criteria. MP analyses were performed in TNT 1.5 (Goloboff et al. 2008; Goloboff and Catalano 2016) using traditional search with characters treated as unordered. MP trees were evaluated by tree length (TL), consistency (CI), and retention indices (RI), and summarized in strict and majority rule consensus trees. Initial fundamental analyses with equal weights were followed by searches with implied weighted schemes (Goloboff 1993) with concavity constant k = 3–25. Standard bootstrapping (Bootstrap support, BS) and symmetric resampling (SR) with 1000 replicates were applied to the unweighted dataset to assess the branch support. Furthermore, Bremer support values (BrS; Bremer 1994) were calculated in TNT for the clades of the unweighted MP tree. Character optimizations were mapped on the strict consensus tree using unambiguous changes, accelerated (ACCTRAN) and delayed (DELTRAN) transformations in WinClada (Nixon 2002).

Maximum likelihood (ML) searches were applied under IQ-Tree 2 software (Minh et al. 2020) with branch support estimated by ultrafast bootstrapping (UFBoot) using 1000 replicates. The best-fit model was selected by ModelFinder (Kalyaanamoorthy et al. 2017) according to Bayesian information criterion (BIC) and applied the *k*-states Markov Mkv (= MK + ASC) model (Lewis 2001) with ascertainment bias correction (ASC), gamma distribution using four discrete rate categories (G4), and equal state frequencies (FQ). Bayesian analyses (BA) were performed in Mr. Bayes 3.2.7 (Ronquist et al. 2012) for two million generations using a stopping value 0.01 of the standard deviation of split frequencies and Mkv model. UFBoot and Bayesian PP values ≥ 95% were interpreted as high nodal support, and ≥ 80% for standard BS values.

Approximately 100 Eurrhacini specimens were examined using an Olympus SZX 12, or Zeiss Stereo Discovery V8 stereoscopic microscopes. Eyes are differentiated into small, medium-sized, and large. In medium-sized eyes, the eye diameter is equal to the interocular distance; in small eyes the eye diameter is less than the interocular distance; in large eyes the eye diameter is greater than the interocular distance. Nine longitudinal elytral costae are distinguished in four strong primary costae and five less elevated alternate secondary costae. Costae and intercostal intervals are numbered from the suture as in other Coleoptera. Dissection of genitalia was made after boiling in 10% KOH solution and followed previous studies (Nascimento and Bocakova 2017). Relative measurements were taken using an ocular micrometer, and dimension measurements (in millimeters) and scale bar insertions were processed by the camera software. Digital photographs were taken using an attached Canon EOS 1100D camera and stacked by QuickPhoto Camera 3.0 microscope software using a Deep Focus 3.3 module. Images were further edited in GIMP 2.10.22 and Adobe Photoshop CS3.

The syntypes of *Calocladonchiriquense* Gorham, 1884 were borrowed from The Natural History Museum (**NHMUK**) in London, U.K., while other material is deposited in the collection of Palacky University Olomouc (**UPOL**), Czech Republic.

## ﻿Results

### ﻿Phylogenetic analyses

**Figure 1.**
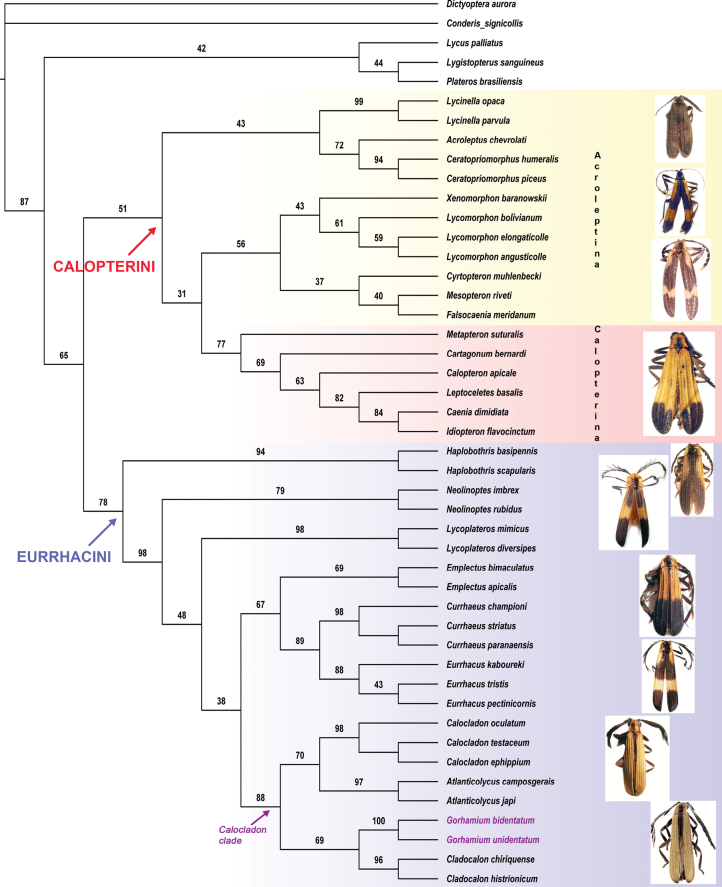
Maximum likelihood phylogeny of Calopterini and Eurrhacini inferred from the morphological dataset using IQ-Tree 2 and the best-fit MK+FQ+ASC+G4 model selected by ModelFinder. Node labels represent ultrafast bootstrap support values.

Different analytical approaches resulted in congruent patterns of major lineages. Our ML tree (Fig. 1) applying the best-fit MK+FQ+ASC+G4 model recovered two distinct clades: Calopterini (UFBoot = 51) and Eurrhacini (UFBoot = 78), although the ultrafast bootstrap support values were low. Inferred internal relationships within Calopterini revealed Calopterina receiving low support (UFBoot = 77), while the Acroleptina were paraphyletic. Basal relationships of Eurrhacini showed a pectinate pattern. The *Calocladon* clade was recovered (UFBoot = 88, Fig. 1), including *Gorhamium* gen. nov. as sister to *Cladocalon*, whereas *Calocladon* was sister to *Atlanticolycus*.

Bayesian analyses of the dataset resulted in trees with low posterior probabilities for Eurrhacini (PP = 0.51), while Calopterini were unsupported (PP = 0.29, Suppl. material 2). The subtribe Calopterina obtained low support (PP = 0.52) and Acroleptina were again found paraphyletic forming two clades. Within the Eurrhacini, the *Calocladon* clade received moderate support (PP = 0.93), whereas support for the crown clade *Gorhamium* gen. nov. + *Cladocalon* was low (PP = 0.61).

Initial unweighted MP analyses resulted in 23 shortest trees (TL = 131, CI = 49.62, RI = 80.7), the strict consensus of which recovered monophyly of Eurrhacini (Suppl. materials 3–5). However, most Calopterini formed a basal multifurcation and the tribe was present only on the majority rule consensus tree (91%, Suppl. material 6). MP analyses found some Bremer support for both Calopterini and Eurrhacini (BrS = 1, Suppl. material 7), while bootstrapping and symmetric resampling showed the clades unsupported (Calopterini - BS = 6, SR = 10; Eurrhacini - BS = 1, SR = 2; Suppl. materials 8, 9). Subsequent implied weighting schemes always resulted in a single identical topology (Suppl. material 10) regardless of the concavity constant applied (k = 3–25). The implied weighted trees showed the Eurrhacini, while Calopterini were broken into the subtribes of Calopterina and Acroleptina.

Phylogenetic relationships within the tribe Eurrhacini revealed the genus *Haplobothris* as the most basal branch in all analyses. The remaining Eurrhacini was strongly supported (UFBoot = 98, PP = 0.98, BrS = 4). MP trees further indicated a bifurcation of *Calocladon* and *Eurrhacus* clades (Suppl. materials 3, 10). While the *Calocladon* clade was supported in all analyses (UFBoot = 88, PP = 0.93, BrS = 3), the latter was paraphyletic in ML and BA trees (Fig. 1, Suppl. material 2). Similarly, relationships within the *Calocladon* clade showed high Bremer support for *Cladocalon* + *Gorhamium* gen. nov. (BrS = 14), whereas the clade received low support in ML and BA trees (UFBoot = 69, pp = 0.6). Our MP analyses also found *Calocladon* + *Atlanticolycus* clade well supported (BrS = 3), but the group received low support in ML analyses (UFBoot = 70) and was broken in Bayesian trees.

### ﻿Taxonomy

#### ﻿ Gorhamium gen. nov.

Taxon classificationAnimaliaColeopteraLycidae

E48A6CB9-FB20-5286-B758-6BEEA6FB4F65

https://zoobank.org/C31BE6D3-296C-45B1-B685-AD85CB34EA65

##### Type species.

*Gorhamiumbidentatum* sp. nov. (by present designation).

##### Diagnosis.

**Figure 2.**
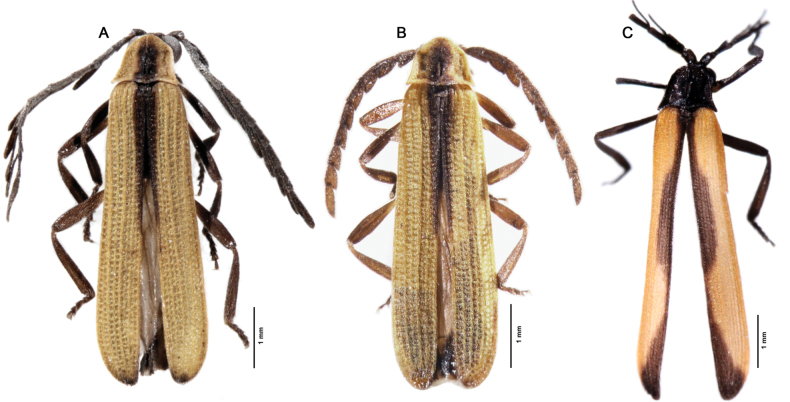
Habitus, dorsal view **A, B***Gorhamiumbidentatum* sp. nov. **A** male **B** female **C***Gorhamiumunidentatum* sp. nov., male.

**Figure 3.**
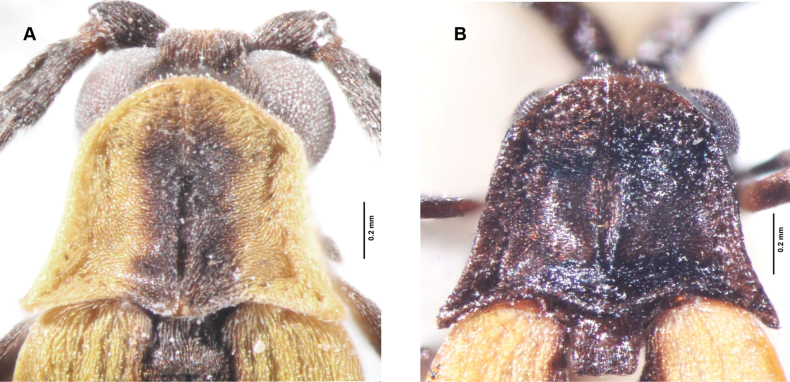
Pronotum **A***Gorhamiumbidentatum* sp. nov., male **B***Gorhamiumunidentatum* sp. nov., male.

**Figure 4.**
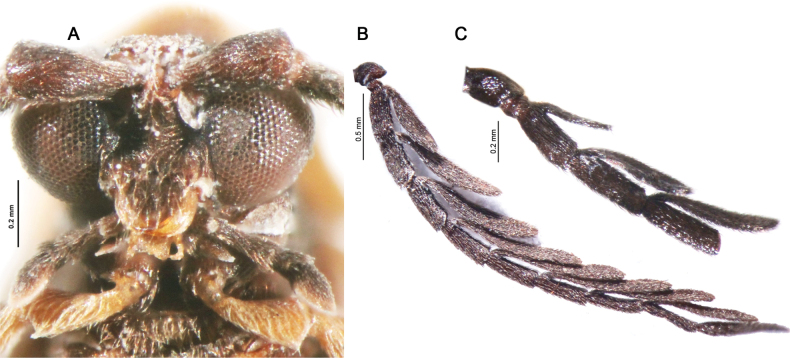
**A** head ventrally **B, C** antenna dorsally **A, B***Gorhamiumbidentatum* sp. nov., male **C***Gorhamiumunidentatum* sp. nov., male.

*Gorhamium* gen. nov. can be distinguished from other Eurrhacini by the combination of the following characters: a) elytra (Fig. 2A–C) with nine longitudinal costae (4 costae in *Haplobothris*); b) pronotum (Fig. 3A, B) wider than long (elongated in *Calocladon*); c) median areola on pronotum slenderly lenticular (slightly wider in *Cladocalon* and *Atlanticolycus*); d) male antennomere 3–10 flabellate (Fig. 4B, C); e) aedeagus with each paramere projected ventrobasally into a slender, medially curved process (d_1_, Fig. 6C), sometimes joining at midline forming an annular bridge (d_3_, Fig. 6G) (also present in *Calocladon*, *Cladocalon*, and *Atlanticolycus*). Among unique features of *Gorhamium* gen. nov. belong: a) base of phallus pointed anchored-shaped (inverted mushroom-shaped), with arcuate arms and a pointed tip (a_1_, Fig. 6E, F), while the base of phallus of *Cladocalon* and *Atlanticolycus* is flat, or rounded (a_2_, Fig. 6A); b) median portion of phallus extending ventrally into oval opening (b, Fig. 6C); c) dorsal edge of phallus hooked (c, Fig. 6D, G); d) internal sac membranous with minute spines distally (e, Fig. 6D, G); e) parameres shorter than 2/3 of phallus (while the parameres are almost as long as phallus in *Atlanticolycus*); f) base of parameres semicircular in cross-section (flattened/ribbon-like in *Cladocalon*); g) apex of parameres denticulate, provided with one or two coarse teeth; h) female genitalia with valvifers as long as coxites and styli combined (Fig. 6H).

##### Description.

Body length: 5.5–6.4 mm, width across the humeri: 1.2 mm. Head partly covered by pronotum from above. Labrum small, mandibles slender, arcuate (Fig. 4A). Maxillary palps 4-segmented, gradually widened distally, palpomere 1 (=P1) at least 3× shorter than P2, P2 longest of all, ~ 2× longer than P4, P3 1.5× shorter than P4, terminal palpomere securiform, apex obliquely rounded (Fig. 4A). Terminal palpomere of labial palps securiform. Pronotum somewhat trapezoidal, with anterior margin produced forward, posterior margin 1.4× wider than median length; lateral margins divergent posterad, with anterior 2/3 almost straight, convergent anteriorly, posterior angles acute; posterior margin bisinuate, medioposterior process almost triangular (Fig. 3A, B); median longitudinal carina on pronotum bifurcating in anterior third, forming very slender, lenticular areola. Scutellum square, apex minutely emarginate medially (Fig. 3A). Elytra subparallel-sided, slender, 4× longer than humeral width (Fig. 2A–C). Each elytron with nine longitudinal costae (4 primary costae and 5 less elevated secondary costae), primary costae 2 and 4 strongly elevated; intercostal intervals with a row of irregular reticulate cells, secondary costae 3 and 4 absent posteriorly. Anterior thoracic spiracles small, tubulate. Legs compressed, trochanters almost triangular (Fig. 4A), as long as third of femur, tibiae straight, their spurs small, covered by pubescence, tarsomeres 1–4 lobed.

**Figure 5.**
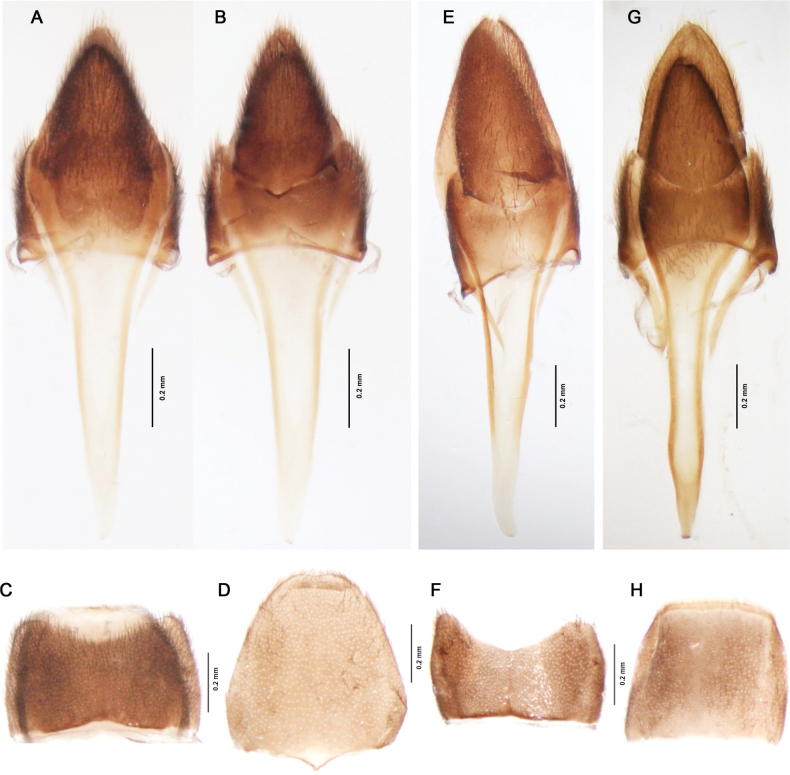
Terminal abdominal segments **A–D***Gorhamiumbidentatum* sp. nov. **E–H***Gorhamiumunidentatum* sp. nov., male **G***Cladocalonchiriquense* (Gorham, 1884) **A, B, E, G** male terminalia (sternum IX and tergum IX–X), **A** – ventral view; **B, E, G** – dorsal view **C** Male sternum and tergum VIII, ventral view **D** female terminal sternum, ventral view. **F**, Male sternum VIII, ventral view **H** Male tergum VIII, dorsal view.

**Figure 6.**
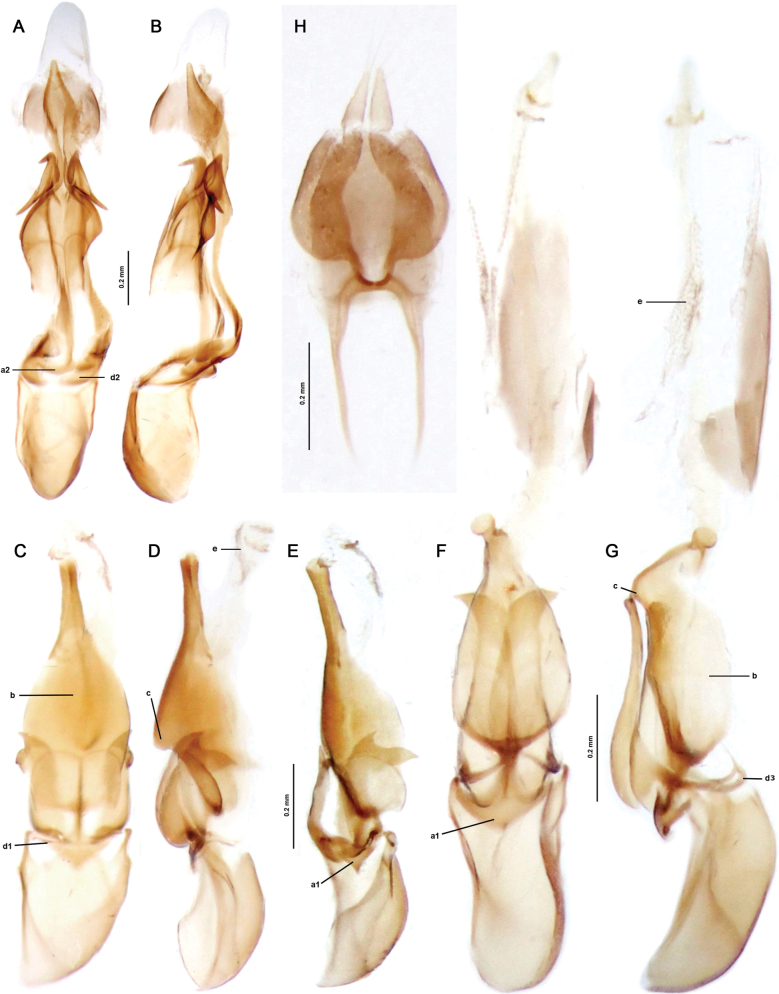
**A–G**, Male genitalia **A, B***Cladocalonchiriquense* (Gorham, 1884) **C–E***Gorhamiumbidentatum* sp. nov. **F–H***Gorhamiumunidentatum* sp. nov. **H** female genitalia of *Gorhamiumbidentatum* sp. nov., ventral view. **A, C, F** ventral view **B, D, G** lateral view **E** ventrolateral view. Abbreviations: a1 – pointed anchor-shaped base of phallus, a2 – flat anchor-shaped base of phallus, b – phallic ventral opening, c – dorsal dent, d1 – arcuate ventrobasal parameral process, d2 – flattened ventrobasal parameral process, d3 – a ring-like ventral bridge (ventrobasal processes medially fused), e – internal sac.

**Male.** Eyes medium-sized to large, eye diameter 1.3–1.7× longer than interocular distance. Antennae reaching beyond elytral midlength, antennomeres 3–10 flabellate, antennal branches flattened, antennomere 1 (=A1) stout, A2 small, transverse, A3 slightly (1.15–1.3×) shorter than A4, A4–A10 subequal in length. Lamellae arise basally, lamella of A3 slightly longer than antennomere body, remaining lamellae considerably longer. Abdominal sternum VIII widely emarginated distally (Fig. 5C, F), emargination shallow, as deep as ¼ of sternum length. Sternum IX elongate, 3.5× longer than wide (Fig. 5A, E), widest in distal quarter, proximal half narrow with lateral margins convergent. Phallus with ventromedial oval opening (b, Fig. 6C, G), base of phallus pointed anchored-shaped, or inverted mushroom-shaped (a_1_, Fig. 6E, F); distal portion of phallus rod-like, apex clavate, dorsal margin hooked (c, Fig. 6D, G); internal sac membranous with minute spines distally (e, Fig. 6G), sometimes also medially. Parameres at most as long as 2/3 of phallus, base of parameres almost semicircular in cross-section; each paramere projected basally in a thin ventral, medially arched, process (d_1_, Fig. 6C), sometimes joining medially in a ring-like bridge (d_3_, Fig. 6G); parameral apex denticulate, provided with one or two coarse teeth. Phallobase slightly asymmetrical, distorted, moderately arched ventrally.

**Female.** Eyes small, interocular distance 1.3× longer than eye diameter, antennae serrate (Fig. 2B). Terminal sternum (IX) simple (Fig. 5D), spiculum gastrale rudimentary, triangular. Ovipositor with valvifers 1.3× longer than coxites (Fig. 6H).

##### Etymology.

The genus is named in honor of H. S. Gorham, the author of chapters on Malacodermata in Biologia-Centrali Americana (Gorham 1880, 1881, 1884), where he described many genera and species of Eurrhacini and Calopterini. The gender is neuter.

##### Distribution.

Panama, Ecuador.

#### ﻿ Gorhamium bidentatum sp. nov.

Taxon classificationAnimaliaColeopteraLycidae

2C643453-BC6F-545E-A4FB-74E4DD6E086A

https://zoobank.org/C1E7CBDC-5EBC-457E-BB8D-5B51E6ACE0F6

Figs 2A, B , 3A , 4A, B , 5A–D , 6C–E, H

##### Type material.

***Holotype*** • male, “Panama, V. de Chiriqui, 25–4000 ft. Champion”, secondary labels - B.C.A. Col. III. (2). Calocladonchiriquense, SYNTYPE - blue-edged circle (BMNH). [Volcan de Chiriqui is now referred to as Volcán Barú].

***Paratypes*** • Panama, same data as for holotype, 1 male, 3 females (BMNH); • “PANAMA, V. de Chiriqui, 2-3000 ft. Champion”, secondary labels - same data as for holotype, SYNTYPE - blue-edged circle, 1 female (BMNH); • “Panama, V. de Chiriqui, 4000–6000 ft. Champion, secondary labels - same data as for holotype, SYNTYPE - blue-edged circle, 3 males (BMNH).

##### Diagnosis.

Pronotum and elytra largely yellow, only median longitudinal stripe on pronotum, basal half of elytral suture, and elytral apex black. Phallus rod-like apically, ventromedial opening oval, widest medially. Parameres shorter than half of phallus, their ventrobasal projects separated (d_1_, Fig. 6C), apex of parameres bidentate, internal sac largely membranous, micro spurs barely visible.

##### Description.

Body length: 5.1–6 mm, width across the humeri: 1.1–1.2 mm. Body dark brown, only anterior pronotal margin, broad sides of pronotum, trochanters, bases of femora, scutellum, and most of elytra yellow (Fig. 2A, B). Sutural stripe in basal half of elytra and apical 1/30 of elytra black. Head largely covered by pronotum. Elytra 4–4.8× longer than humeral width (Fig. 2A). Primary costae 2 and 4 and basal quarter of primary costa 3 more elevated. Reticulate cells irregular, secondary costae 3 and 4 present only basally.

**Male.** Eyes large, hemispherically prominent, eye diameter 1.5–1.7× longer than interocular distance. Antennae with antennomere 3 (=A3) 1.15× shorter than A4, A4–A10 subequal in length; antennal branches flattened, lamella of A3 1.7× longer than antennomere length, remaining lamellae considerably longer, ~ 2.4× longer than antennomere length (Fig. 4B). Abdominal sternum VIII with a broad, shallow emargination distally (up to 1/5 of sternum length), its proximal margin minutely emarginated up to 1/10 of sternum length (Fig. 5C). Tergum X small, only 1.3× longer than preceding sternum IX on the sides (Fig. 5B). Phallus rod-like in distal 1/3, slightly widened apically, with a dorsal hook in median portion (c, Fig. 6D) and large ventral opening widest medially (b, Fig. 6C). Parameres moderately shorter than half of phallus, each with two coarse teeth apically, ventrobasal parameral protrusions slender, medially separated by 1/3 of phallic width (Fig. 6C).

**Female.** Eyes small, eye diameter 1.3× shorter than interocular distance. Antennae serrate (Fig. 2B). Terminal sternum with spiculum gastrale rudimentary, triangular to slightly pointed (Fig. 5D). Ovipositor elongate (Fig. 6H), valvifers rod-like, 1.4× longer than coxites, basally coalescent. Coxites medially distant, their base and apex closer, styli as long as half of coxites.

##### Etymology.

Named after the shape of apical portion of parameres.

##### Distribution.

Panama.

#### ﻿ Gorhamium unidentatum sp. nov.

Taxon classificationAnimaliaColeopteraLycidae

C6E98B65-EB05-5292-A981-4D46CFC92555

https://zoobank.org/8CEF51B9-4C36-4654-BEB4-B3BFA4F63DC3

Figs 2C , 3B , 4C , 5E–H , 6F, G

##### Type material.

***Holotype*** • male, “Ecuador, 50 km SW Quito, San Francisco de las Pampas, Otonga res., 1500 m, 0°25'S, 79°00'W, 5–6.Dec 2010, Bolm lgt.“ (UPOL).

##### Diagnosis.

Pronotum black. Elytra bicolor orange-black with suture, longitudinal median oval spot, and triangular apical spot black. Phallus ball-shaped apically, ventromedial opening widest in basal third. Apex of each paramere fitted with a sharp laterally projected tooth, internal sac with a series of diminutive teeth (e, Fig. 6G).

##### Description.

Body length: 6.4 mm, width across the humeri: 1.2 mm. Body black, only elytral sidebars orange (with whole suture, longitudinal median oval spot and triangular spot in apical quarter black, remaining sidebars orange (Fig. 2C). Head mostly hidden by pronotum in dorsal view. Elytra slender, 4.5× longer than humeral width (Fig. 2C); primary costae 2 and 4 and basal 1/5 of primary costa 3 elevated; reticulate cells oval, strongly irregular, secondary costae 3 and 4 diminishing apically.

**Male.** Eyes medium-sized, interocular distance 1.3× longer than eye diameter (Fig. 3B). Antennae with antennomere 3 (= A3) 1.3× shorter than A4, A4–A10 subequal in length; antennal branches flattened, considerably lengthening medially, A3 lamella 1.2× longer than antennomere A3 length, A4 lamella 1.35× longer than A4 length, A5 lamella 1.5× longer than A5 length (Fig. 4C). Abdominal sternum VIII widely emarginated in distal third (Fig. 5F), its proximal margin almost straight. Tergum X elongate, 1.7× longer than sternum IX on the sides (Fig. 5E). Phallus bent ventrally in distal 1/3, constricted subapically, apex ball-shaped; ventromedial opening widest in basal quarter (b, Fig. 6G); dorsal hook shifted in distal quarter (c, Fig. 6G). Parameres as long as 2/3 of phallus, with a single, laterally projected, apical tooth; ventrobasal parameral protrusions slender, joined medially in a ring-like bridge (d_3_, Fig. 6G).

**Female.** Unknown.

##### Etymology.

The specific name refers to the single sharp tooth at the apex of each paramere.

##### Distribution.

Ecuador.

### ﻿Key to genera of Eurrhacini

**Table:** 

1	Each elytron with only 4 longitudinal costae, secondary costae absent	***Haplobothris* Bourgeois, 1879**
–	Each elytron with 9 longitudinal costae, alternate costae strong, more elevated	**2**
2	Pronotum with a median longitudinal carina, areola absent, or at most slot-like	**3**
–	Pronotum with longitudinal carinae forming median longitudinal areola	**4**
3	Median longitudinal areola on pronotum absent, posterior pronotal margin with prominent medioposterior protrusion covering whole scutellum, male antennae flabellate	***Lycoplateros* Pic, 1922**
–	Median longitudinal areola on pronotum slot-like, basal margin of pronotum almost straight in median portion, scutellum visible, male antennae serrate	***Neolinoptes* Nascimento & Bocakova, 2017**
4	Aedeagus trilobate, parameres often shortened, but separate from the phallus, basal portion of each paramere with an arcuate ventral protrusion, usually joining medially in a ring-like bridge (character 45, state 1)	**7**
–	Aedeagus unilobed, parameres either absent, or strongly shortened and coalescent with phallus, sometimes with remnants of sutures dorsally	**5**
5	Male genitalia with phallobase not fused to phallus and parameres, terminal maxillary palpomere enlarged, 1.8× longer than palpomere 2 (P2); parameres entirely integrated into the widened basal 1/10–1/3 of tubular phallus, posterior trochanters spinose	***Eurrhacus* Waterhouse, 1879**
–	Male genitalia with phallobase fused to phallus and parameres (if present), terminal maxillary palpomere small, 1.3–1.6× shorter than P2	**6**
6	Phallus and phallobase ventrally coalescent to parameres, basal 3/5 of phallus with integrated parameres conical, parameres dorsally visible, slightly folded. Terminal maxillary palpomere 1.3× shorter than P2, posterior trochanters triangular	***Emplectus* Erichson, 1847**
–	Parameres absent, phallus S-shaped, basally fused to median portion of phallobase, terminal maxillary palpomere 1.6× shorter than P2	***Currhaeus* Nascimento, Bressan & Bocakova, 2020**
7	Pronotum ~ 1.3× longer than wide; apical half of phallus strongly curved ventrally, parameres short, as long as 1/3 of phallus; base of phallus sharply triangular (character 47, state 1), integrated to dorsobasal portion of parameres; phallobase elongate, as long as 2/3 of phallus	***Calocladon* Gorham, 1881**
–	Pronotum wider than long, base of phallus anchor-shaped (inverted mushroom-shaped) (Fig. 6A–G)	**8**
8	Primary costa 3 usually joined to primary costa 2 in distal 1/3–1/4 of elytra. Parameres almost as long as phallus, laterally compressed, connected basally by a strong annular ventral bridge, apex rounded	***Atlanticolycus* Nascimento & Bocakova, 2023**
–	Primary costa 3 almost fully developed, not joining to primary costa 2. Parameres shorter than the phallus by at least a quarter of the length, distal half flattened, with ventrobasal projects either strongly flattened (Fig. 6A, B), or slender (d_1_, Fig. 6C), sometimes fused forming ventral bridge (d_3_, Fig. 6G)	**9**
9	Parameres flattened, ribbon-like, L-shaped in lateral view, apex with basally-oriented hooks, ventrobasal parameral projects flattened, sometimes constituting a ventral bridge, base of phallus more or less flat anchor-shaped (inverted mushroom-shaped)	***Cladocalon* Nascimento & Bocakova, 2022**
–	Parameres basally semicircular in cross-section, apex of parameres with 1 or 2 laterodistal teeth. Ventrobasal parameral projects, or ventral bridge very slender, base of phallus pointed anchor-shaped, or inverted mushroom-shaped (character 47, state 1)	***Gorhamium* gen. nov.**

## ﻿Discussion

### ﻿The Calopterini

Support for a monophyletic origin of Calopterini and the subtribe Calopterina has been confirmed by previous (Bocakova 2005; Nascimento et al. 2020; Ferreira et al. 2023) and our current (this study) morphology-based analyses. However, the formerly recovered Acroleptina, comprising all neotenic calopterins, is now predominantly paraphyletic (Table 2) and split into two lineages.

**Table 2.** Support for major Calopterini and Eurrhacini lineages (Bocakova 2005; Bocak and Bocakova 2008; this study). Branch support values are based on ultrafast bootstrapping (UFBoot) for maximum likelihood (ML) and posterior probabilities (PP) for Bayesian analyses (BA). For maximum parsimony (MP) analyses, clade percentages on the majority-rule consensus tree (MRCT), standard bootstrapping (BS) and symmetric resampling (SR) values are given. Abbreviations: P – paraphyletic or polyphyletic; NW – no weights, IW – implied weights.

Tree search procedures	ML	BA	MP	MP	MP	MP
			NW	NW	NW	IW
	UFBoot	PP	MRCT	BS	SR	MRCT
Calopterini + Eurrhacini	65	38	**100**	22	24	**100**
Calopterini	51	29	**91**	6	10	P
Calopterina	77	52	P	10	16	**100**
Acroleptina	P	P	**100**	P	P	**100**
Eurrhacini	78	51	**100**	1	2	**100**
*Calocladon* clade	**88**	**93**	**100**	49	55	**100**
*Eurrhacus* clade	P	P	**100**	P	P	**100**

### ﻿The Eurrhacini

Consistent with our results, previous analyses supported the Eurrhacini and showed *Haplobothris* as the deepest branch. The initial trees (Bocakova 2005; Nascimento et al. 2020) further implied an early separation of *Calocladon*. However, after the inclusion of *Xenomorphon*, an enigmatic anelytrous beetle male (Ferreira et al. 2023), *Calocladon* was recovered as a crown group being sister to *Lycoplateros*, although support values were low. By contrast, our results (Fig. 1) have indicated the *Calocladon* clade is sister to *Emplectus* + *Eurrhacus* + *Currhaeus* clade, whereas *Lycoplateros* is recovered as one of early Eurrhacini branches.

### ﻿The *Calocladon* clade

Our updated dataset is the first to include the recently described *Atlanticolycus* (Brazil), *Cladocalon* (Mexico, Guatemala, and Panama), and *Gorhamium* gen. nov. (Panama, Ecuador) proposed here. The analyses show *Calocladon* and the three closely related genera constitute a highly supported clade (UFBoot = 88, pp = 0.93).

Members of the *Calocladon* clade share two unambiguous synapomorphies (Suppl. materials 3–5), particularly the convergent ventrobasal projections on the parameres that often fuse medially into a ventral bridge (character 45, state 1; Fig. 6G, d_3_). The character is present in all genera, although the length and thickness of these projections varies. While *Calocladon* and *Atlanticolycus* have the strongly developed ventral bridge of the parameres, the ventrobasal parameral projects are often shorter and less pronounced in the *Cladocalon* + *Gorhamium* clade. The second unambiguous synapomorphy of the *Calocladon* clade is the strong, sharply triangular, or inverted mushroom-shaped base of phallus (character 47, state 1). Furthermore, *Cladocalon* has the characteristic L-shaped parameres. The feature is also present in *Gorhamiumunidentatum* sp. nov. (Fig. 6G), while it is only indicated in *G.bidentatum* sp. nov. (Fig. 6C, D). Genera *Cladocalon*, *Atlanticolycus*, and *Gorhamium* gen. nov. also share several external characters as flabellate antennae in males, transversely trapezoidal pronotum with lenticular median areola (areola absent, replaced by median longitudinal carina in *Lycoplateros* and *Neolinoptes*), and each elytron with nine longitudinal costae (i.e., secondary less elevated alternate costae present). Conversely, secondary costae are absent in *Haplobothris* (each elytron with only four longitudinal costae). While in *Eurrhacus* and *Lycoplateros* primary costae 1 and 3 are strongly elevated, the genera of the *Calocladon* clade have primary costae 1 and 3 only slightly thicker compared to primary costae 2 and 4. These features are also shared by *Calocladon*, except for its characteristic elongated pronotum and considerably more slender median areola.

## Supplementary Material

XML Treatment for Gorhamium

XML Treatment for Gorhamium bidentatum

XML Treatment for Gorhamium unidentatum

## ﻿Additional information

### Author ORCIDs

Elynton Alves Nascimento https://orcid.org/0000-0002-9071-2823

Milada Bocakova https://orcid.org/0000-0002-2507-0887

### Data availability

All of the data that support the findings of this study are available in the main text or Supplementary Information.

## Supplementary materials

Supplementary material 1 List of morphological characters Data type docx Explanation note List of morphological characters (adapted from Bocakova 2005; Nascimento et al. 2020; Ferreira et al. 2023). Characters 6 and 42 were edited. Characters 12, 17, 21, and 30 include new character states. Characters 44–51 were newly added. https://binary.pensoft.net/file/1063781This dataset is made available under the Open Database License (http://opendatacommons.org/licenses/odbl/1.0/). The Open Database License (ODbL) is a license agreement intended to allow users to freely share, modify, and use this Dataset while maintaining this same freedom for others, provided that the original source and author(s) are credited.Elynton Alves do Nascimento, Milada Bocakova

Supplementary material 2 Bayesian phylogeny of Calopterini and Eurrhacini Data type tif Explanation note Bayesian phylogeny of Calopterini and Eurrhacini inferred from morphological data, node labels represent posterior probabilities. https://binary.pensoft.net/file/1063782This dataset is made available under the Open Database License (http://opendatacommons.org/licenses/odbl/1.0/). The Open Database License (ODbL) is a license agreement intended to allow users to freely share, modify, and use this Dataset while maintaining this same freedom for others, provided that the original source and author(s) are credited.Elynton Alves Nascimento, Milada Bocakova

Supplementary material 3 Strict consensus of 23 parsimony trees of Calopterini and Eurrhacini using equal weights Data type tif Explanation note Unambiguous character changes mapped on branches in WinClada, black circles represent nonhomoplasious changes, white circles homoplasious changes. Circles are labelled with small character numbers above and character states below. https://binary.pensoft.net/file/1063783This dataset is made available under the Open Database License (http://opendatacommons.org/licenses/odbl/1.0/). The Open Database License (ODbL) is a license agreement intended to allow users to freely share, modify, and use this Dataset while maintaining this same freedom for others, provided that the original source and author(s) are credited.Elynton Alves Nascimento, Milada Bocakova

Supplementary material 4 Strict consensus of 23 unweighted parsimony trees, fast optimization using ACCTRAN Data type tif Explanation note Fast optimization using accelerated (ACCTRAN) transformations mapped on branches in WinClada, black circles represent non-homoplasious changes, white circles homoplasious changes. Circles are labelled with small character numbers above and character states below. https://binary.pensoft.net/file/1063784This dataset is made available under the Open Database License (http://opendatacommons.org/licenses/odbl/1.0/). The Open Database License (ODbL) is a license agreement intended to allow users to freely share, modify, and use this Dataset while maintaining this same freedom for others, provided that the original source and author(s) are credited.Elynton Alves Nascimento, Milada Bocakova

Supplementary material 5 Strict consensus of 23 unweighted parsimony trees, fast optimization using DELTRAN Data type tif Explanation note Slow optimization using delayed (DELTRAN) transformations mapped on branches in WinClada, black circles represent nonhomoplasious changes, white circles homoplasious changes. Circles are labelled with small character numbers above and character states below. https://binary.pensoft.net/file/1063785This dataset is made available under the Open Database License (http://opendatacommons.org/licenses/odbl/1.0/). The Open Database License (ODbL) is a license agreement intended to allow users to freely share, modify, and use this Dataset while maintaining this same freedom for others, provided that the original source and author(s) are credited.Elynton Alves Nascimento, Milada Bocakova

Supplementary material 6 The majority-rule consensus of the 23 MP trees from the initial equal weights parsimony analysis of Calopterini and Eurrhacini Data type tif https://binary.pensoft.net/file/1063786This dataset is made available under the Open Database License (http://opendatacommons.org/licenses/odbl/1.0/). The Open Database License (ODbL) is a license agreement intended to allow users to freely share, modify, and use this Dataset while maintaining this same freedom for others, provided that the original source and author(s) are credited.Elynton Alves Nascimento, Milada Bocakova

Supplementary material 7 Bremer support values mapped on the strict consensus of 23 parsimony unweighted trees of Calopterini and Eurrhacini Data type tif https://binary.pensoft.net/file/1063787This dataset is made available under the Open Database License (http://opendatacommons.org/licenses/odbl/1.0/). The Open Database License (ODbL) is a license agreement intended to allow users to freely share, modify, and use this Dataset while maintaining this same freedom for others, provided that the original source and author(s) are credited.Elynton Alves Nascimento, Milada Bocakova

Supplementary material 8 Branch support using standard bootstrapping applied on the unweighted Calopterini-Eurrhacini dataset Data type tif https://binary.pensoft.net/file/1063788This dataset is made available under the Open Database License (http://opendatacommons.org/licenses/odbl/1.0/). The Open Database License (ODbL) is a license agreement intended to allow users to freely share, modify, and use this Dataset while maintaining this same freedom for others, provided that the original source and author(s) are credited.Elynton Alves Nascimento, Milada Bocakova

Supplementary material 9 Branch support using symmetric resampling applied on the unweighted Calopterini-Eurrhacini dataset Data type tif https://binary.pensoft.net/file/1063789This dataset is made available under the Open Database License (http://opendatacommons.org/licenses/odbl/1.0/). The Open Database License (ODbL) is a license agreement intended to allow users to freely share, modify, and use this Dataset while maintaining this same freedom for others, provided that the original source and author(s) are credited.Elynton Alves Nascimento, Milada Bocakova

Supplementary material 10 The single implied weighted parsimony tree using TNT and the concavity constant in the range k = 3–25 Data type tif https://binary.pensoft.net/file/1063790This dataset is made available under the Open Database License (http://opendatacommons.org/licenses/odbl/1.0/). The Open Database License (ODbL) is a license agreement intended to allow users to freely share, modify, and use this Dataset while maintaining this same freedom for others, provided that the original source and author(s) are credited.Elynton Alves Nascimento, Milada Bocakova
