# Supplementary material for: ﻿Phylogenetic analysis reveals a new net-winged beetle genus of Eurrhacini (Coleoptera, Lycidae) from the Pacific slopes of Central America and Ecuador
Source: Zookeys. 2024 Jun 6;1204:241–59. doi: 10.3897/zookeys.1204.114932 (PMC11176813; doi:10.3897/zookeys.1204.114932)
Supplement: Supplementary material 1 — List of morphological characters [file zookeys-1204-241_article-114932__-s001.docx]

**Supplementary material 1**

List of morphological characters (adapted from Bocakova 2005, Nascimento et al. 2020, and Ferreira et al. 2023). Characters 6 and 42 were edited. Characters 12, 17, 21, and 30 include new character states. Characters 44–51 were newly added.

1. Pronotum, transverse costae: present (0); absent (1).

2. Pronotum, median longitudinal areola: absent (0); present at least in basal half (1).

3. Pronotum, carinae forming longitudinal areola: divergent backward, forming slender triangle (0); convergent backward (1).

4. Female terminal sternum, spiculum gastrale: present (0); absent (1).

5. Number of elytral costae: nine (0); three to four (1).

6. Secondary elytral costae: fully developed, straight and stout (0); weakened, strongly irregular, punctuated, or shortened (1). 7. Each elytron with: 4 primary costae (0); at most 3 primary costae (1).

8. Male antennae: filiform to serrate (0); flabellate, pectinate (1);

9. Male hind trochanters: subtriangular, strongly wider than base of femur (0); slender, elongate, slightly wider than base of femur (1); subtriangular, apex protruding in thorn (2).

10. Male terminal sternum: short, proximal tip slightly exceeding attachments of penultimate tergum (the exceeding portion at most as long as the tergum body without attachments (0); long, its proximal tip considerably exceeding attachments of penultimate tergum, with the exceeding portion almost as long as the penultimate tergum including attachments, or longer (1).

11. Aedeagus, dorsomedial portion of phallus in lateral view: plain (0); emarginate, provided with two phallic tubercles in the points of parameral apex (1).

12. Parameres, apex, subapical ventrally oriented thorns: absent, apex simple, rounded (0); 1–2 small, acute, as long as 1/10-1/6 of the length of paramere (1); 1–2 medium-sized, widely obtuse, as long as 1/10 of the length of paramere (2); 1–2 large and long, acute, as long as 1/4-1/3 of the length of paramere (3); with a series of minute apical/subapical thorns (4).

13. Aedeagus, internal sac of phallus: membranous, hardly visible, without sclerites or thorns (0); membranous, easily visible, without sclerites or thorns (1); membranous easily visible with sclerites or thorns (2).

14. Distal portion of phallus: almost cylindrical (0); dorsoventrally flattened (1).

15. Aedeagus, parameres: present (0); absent (1).

16. Aedeagus parameres: not mutually connected ventrally (0); ventrobasally fused (1).

17. Phallus, ventrodistal opening: small, as a point (0); large, almost as long as distal half (1); large, shifted medially, as long as half of phallus (2).

18. Parameres dorsally: at least partly fused or closely attached to each other (0); not mutually connected, loose (1).

19. Ovipositor, valvifers: separate (0); basally fused forming medial bridge (1).

20. Ovipositor, valvifers: long or medium sized (0); extremely short, as projections only (1).

21. Ovipositor, inner margin of coxites: almost straight (0); slightly emarginate medially, slightly extended basally, inner plate

not developed (1); strongly emarginate medially, provided with inner plate basally (2); strongly emarginate medially, connected by basal projections (3).

22. Valvifers, basal bridge: absent to short (0); long (at least as long as 1/3 of valvifer length) and narrow (1).

23. Female genital ducts, vaginal glands: intimately attached to vagina (0); broadly attached to vagina, almost heart shaped (1).

24. Vaginal glands: elongate (0); globular (1).

25. Phallobase: symmetrical laterally (0); slightly distorted clockwise (1).

26. Terminal maxillary palpomere: securiform (0); apically pointed (1).

27. Aedeagus, phallus: almost straight, slightly bent laterally (0); strongly (right angle) bent ventrally in basal 1/4 (1).

28. Aedeagus, phallus dorsally: straight to arcuate ventrally, or emarginate in distal half (0); emarginate in distal 1/4 (1).

29. Aedeagus, phallobase: elongate (0); annuliform (1).

30. Aedeagus, orifice of phallus: placed on apical surface (0); obliquely inserted in apical emargination (1); oval orifice shifted ventromedially, apex tubuliform (2).

31. Female genitalia, coxites: gradually tapering apex (0); strongly constricted in distal 1/3 (1).

32. Female genitalia, basal valviferal plate: parallel to widened distally, not emarginate behind attachment of valvifers (0); obliquely narrowed distally, emarginate behind attachment of valvifers (1).

33. Pronotum, median longitudinal areola: long, as long as 2/3–4/5 of prononal length (0), short, as long as basal half (1).

34. Reticulate cells on elytra: almost square, up to 1.2× longer than wide, or irregular (0); strongly transverse, about 1.5–2×longer than wide (1).

35. Anterior margin of pronotum: transverse, or slightly arcuate (0); triangular (1); trapezoidal (2).

36. Phallus in ventral view: symmetrical (0); slightly asymmetrical, moderately arcuate laterally (1); asymmetrical, S-shaped, twisted clockwise (2).

37. Aedeagus, base of phallus: slender (0); strongly widened, almost bulbous (1).

38. Posterior margin of pronotum: almost straight (0); with a large medioposterior projection usually covering whole scutellum (1).

39. Aedeagus, phallobase: plate like (0); ladle shaped to hood shaped (1).

40. Elytra: present (0); absent (1).

41. Pronotum, overall shape: Subpentagonal (0); Trapezoidal (1); Rectangular (2).

42. Pronotum, median longitudinal carina present at least in anterior third to half: Absent (0); Present (1).

43. Pronotum, median longitudinal carina present at least in anterior half, development. Strongly developed and distinctly visible (0); Weakly developed (1).

44. Elytra, primary costa 3: Almost fully developed, not joining to primary costa 2 (0); Absent in distal 1/3-1/4, joining to primary costa 2 (1).

45. Aedeagus, ventrobasal protrusion of each paramere (sometimes joining medially in a ventral bridge): As long as wide, joining medially (0); Slender, at least 3× longer than wide (1).

46. Aedeagus, parameres, slender ventrobasal protrusions: Separate (0); Joining in ventral bridge (1).

47. Aedeagus, base of phalus dorsally: Weakly sclerotized (0); Strongly sclerotized (triangular, or anchor-shaped) (1).

48. Aedeagus, strongly sclerotized dorsobasal portion of phalus: Sharply triangular (0); Anchor-shaped, basally pointed (1); Anchor-shaped, basally flat, or rounded (2).

49. Aedeagus, dorsal edge of phallus: Simple (0); Hooked (1).

50. Aedeagus, parameres basally: Semicircular in cross-section (0); Flattened/ribbon-like (1).

51. Aedeagus, parameres, 1-2 long apical thorns: Minute to absent (0); very slender, hooked, oriented ventro-proximally (1); large, oriented ventro-distally (2).
