# Supplementary figures and images for: ﻿Phylogenetic analysis reveals a new net-winged beetle genus of Eurrhacini (Coleoptera, Lycidae) from the Pacific slopes of Central America and Ecuador
Source: Zookeys. 2024 Jun 6;1204:241–59. doi: 10.3897/zookeys.1204.114932 (PMC11176813; doi:10.3897/zookeys.1204.114932)

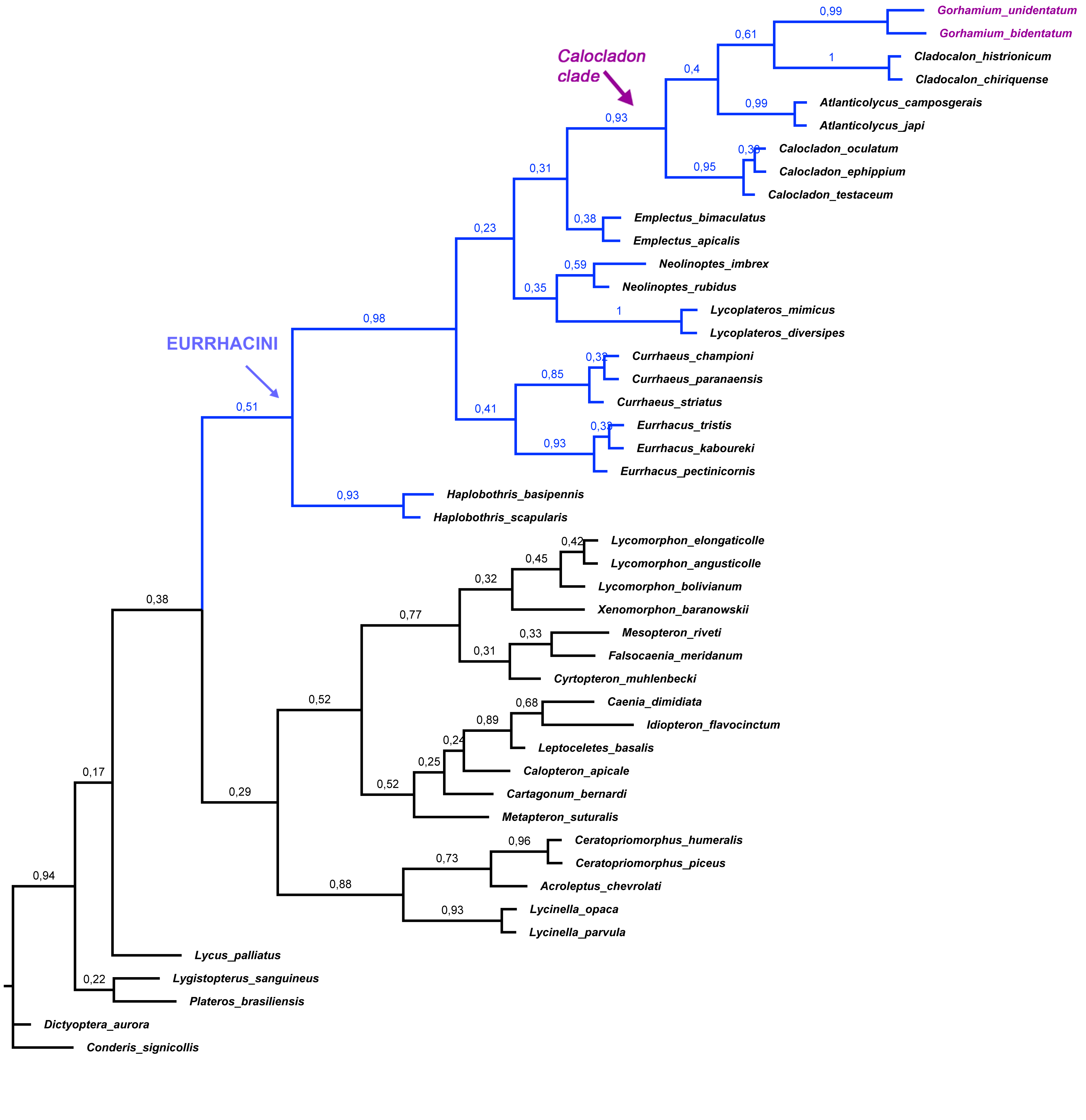

Supplement: Supplementary material 2 — Bayesian phylogeny of Calopterini and Eurrhacini [file zookeys-1204-241_article-114932__-s002.tif]

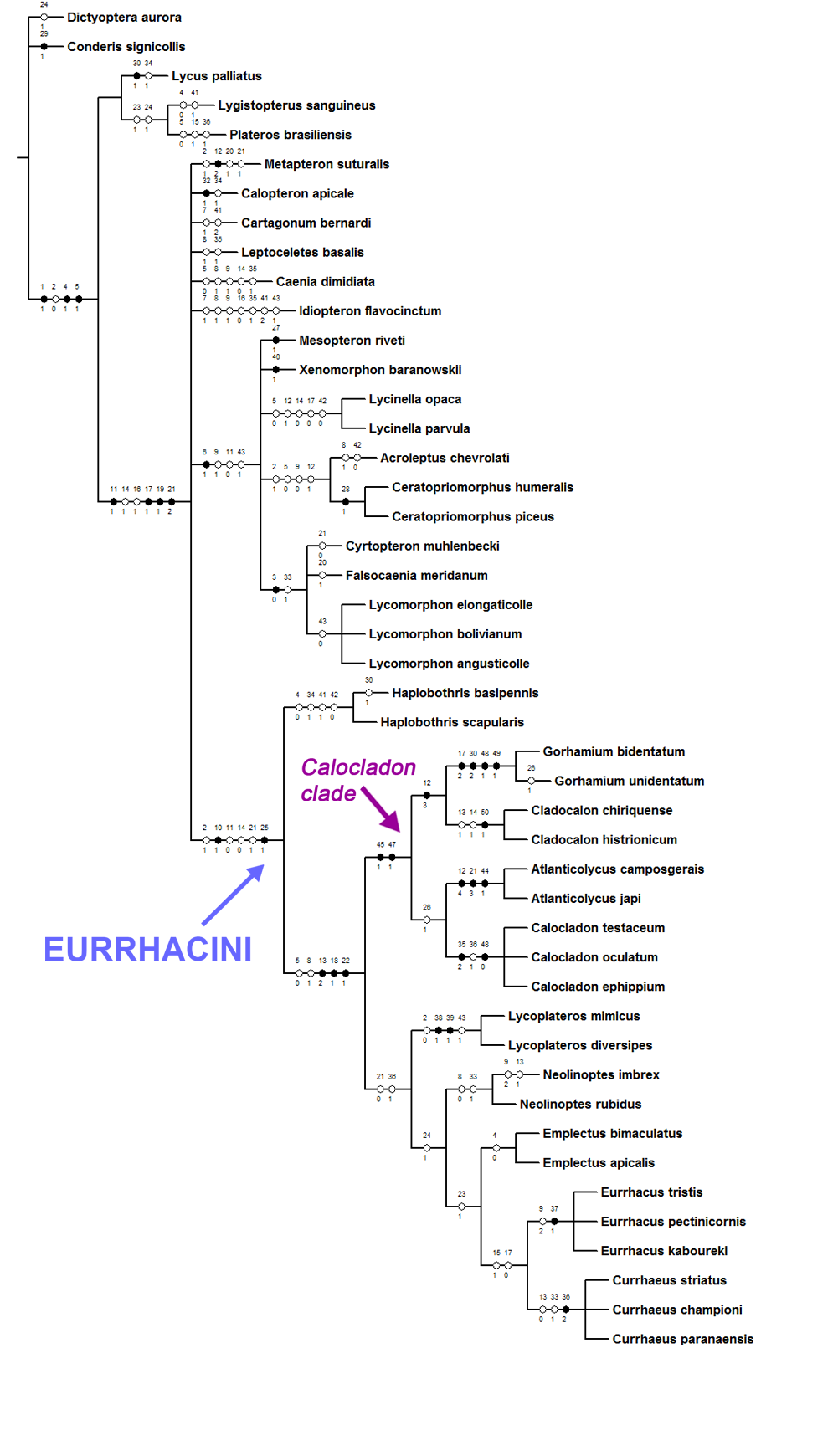

Supplement: Supplementary material 3 — Strict consensus of 23 parsimony trees of Calopterini and Eurrhacini using equal weights [file zookeys-1204-241_article-114932__-s003.tif]

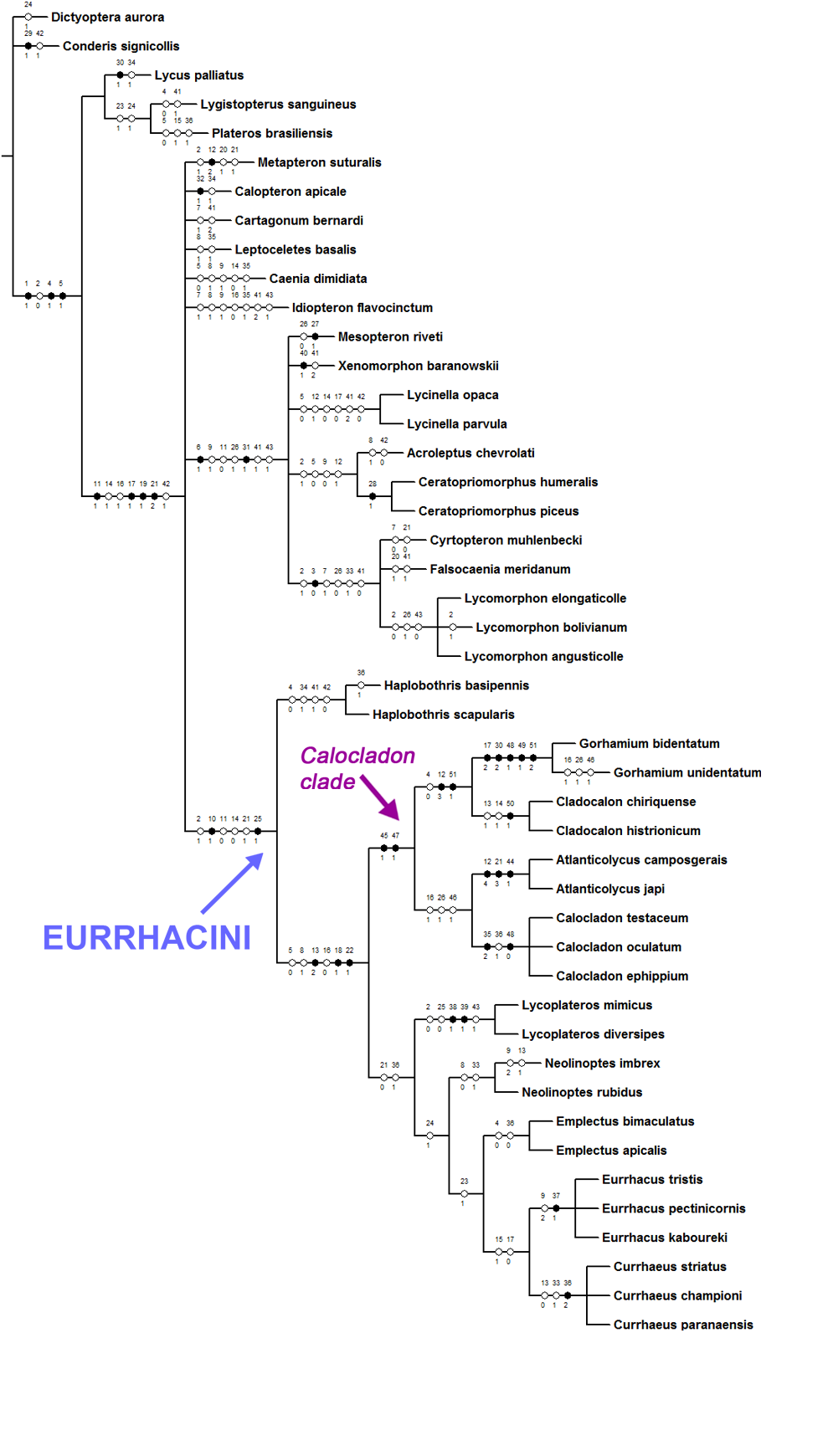

Supplement: Supplementary material 4 — Strict consensus of 23 unweighted parsimony trees, fast optimization using ACCTRAN [file zookeys-1204-241_article-114932__-s004.tif]

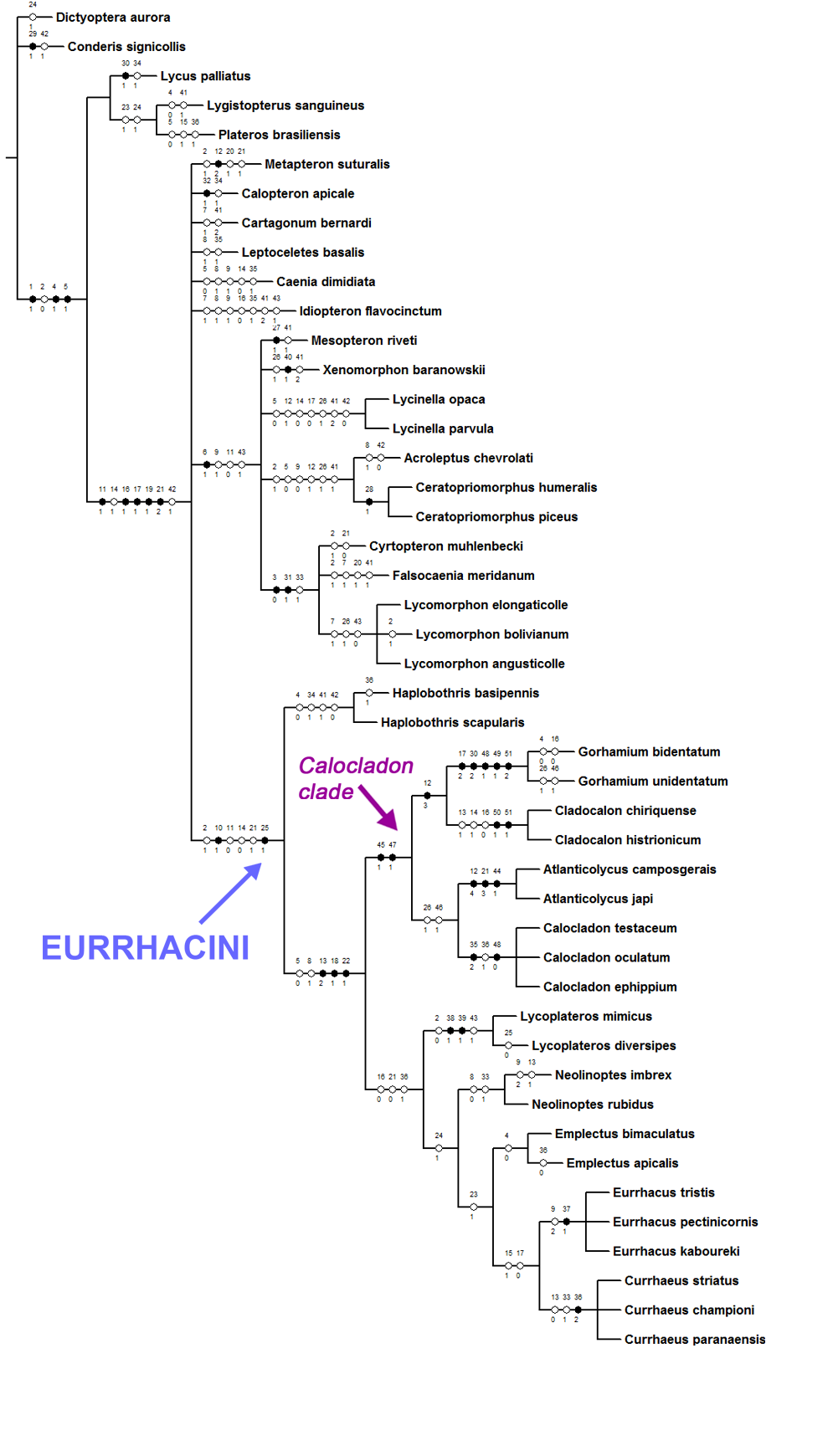

Supplement: Supplementary material 5 — Strict consensus of 23 unweighted parsimony trees, fast optimization using DELTRAN [file zookeys-1204-241_article-114932__-s005.tif]

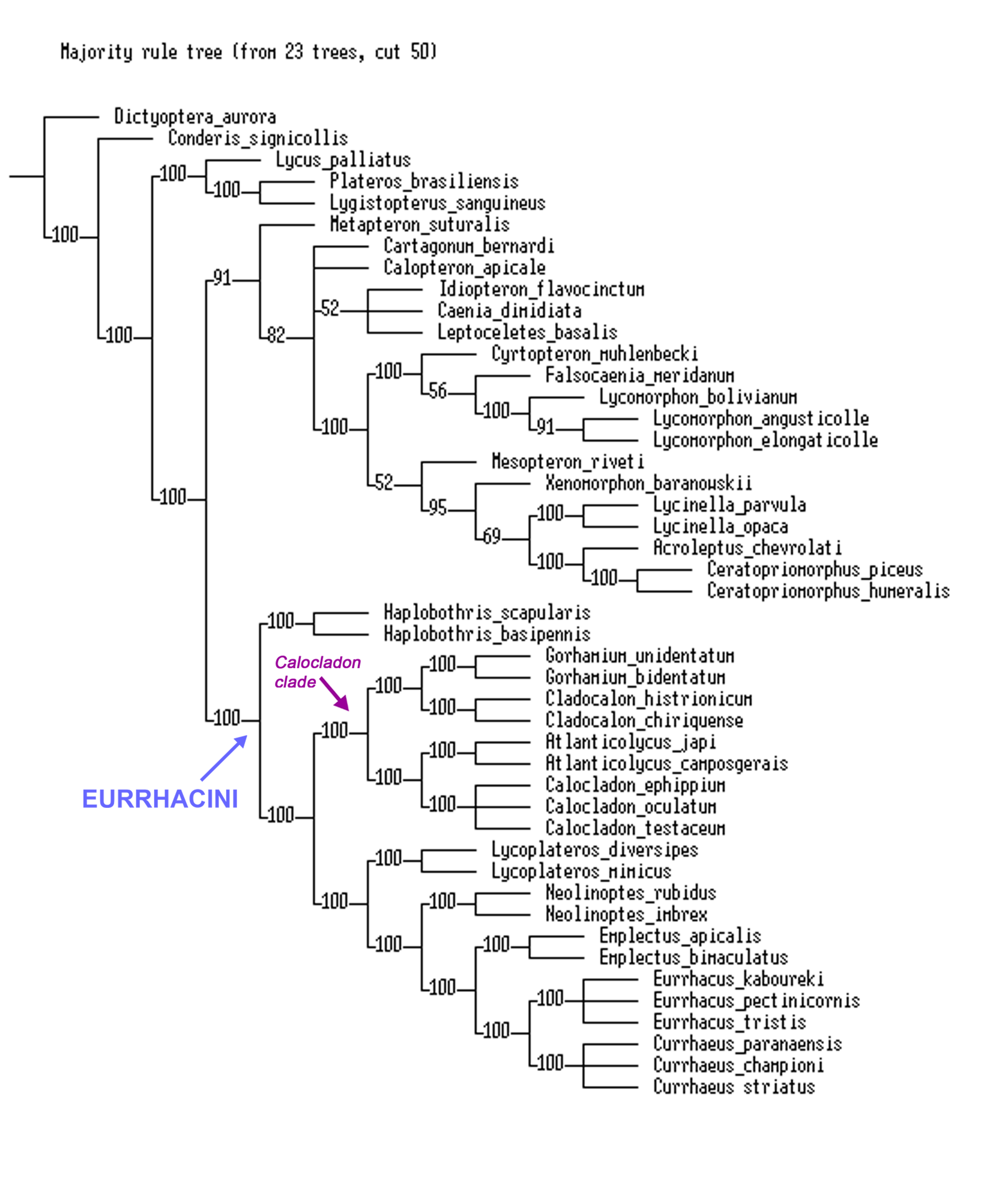

Supplement: Supplementary material 6 — The majority-rule consensus of the 23 MP trees from the initial equal weights parsimony analysis of Calopterini and Eurrhacini [file zookeys-1204-241_article-114932__-s006.tif]

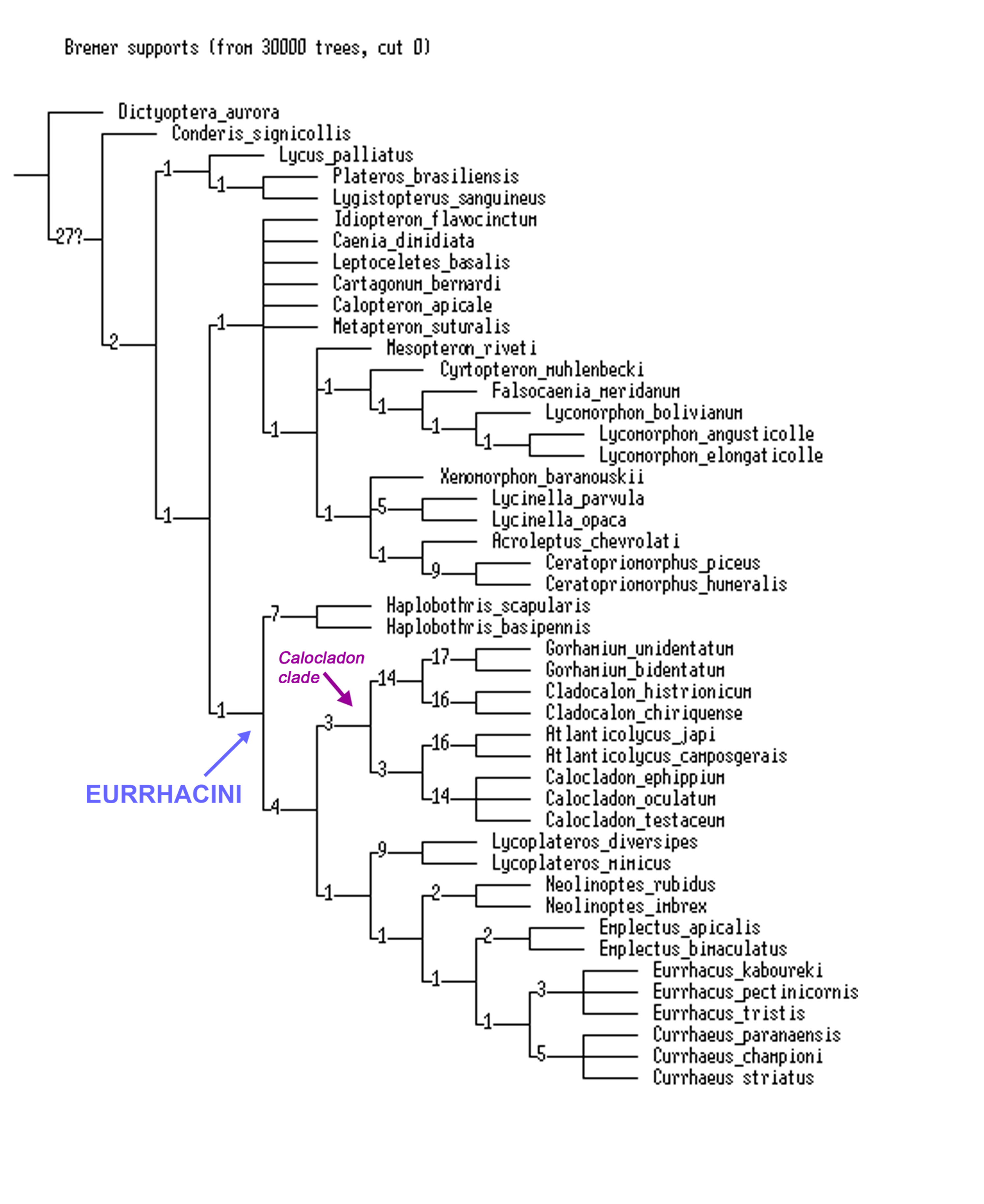

Supplement: Supplementary material 7 — Bremer support values mapped on the strict consensus of 23 parsimony unweighted trees of Calopterini and Eurrhacini [file zookeys-1204-241_article-114932__-s007.tif]

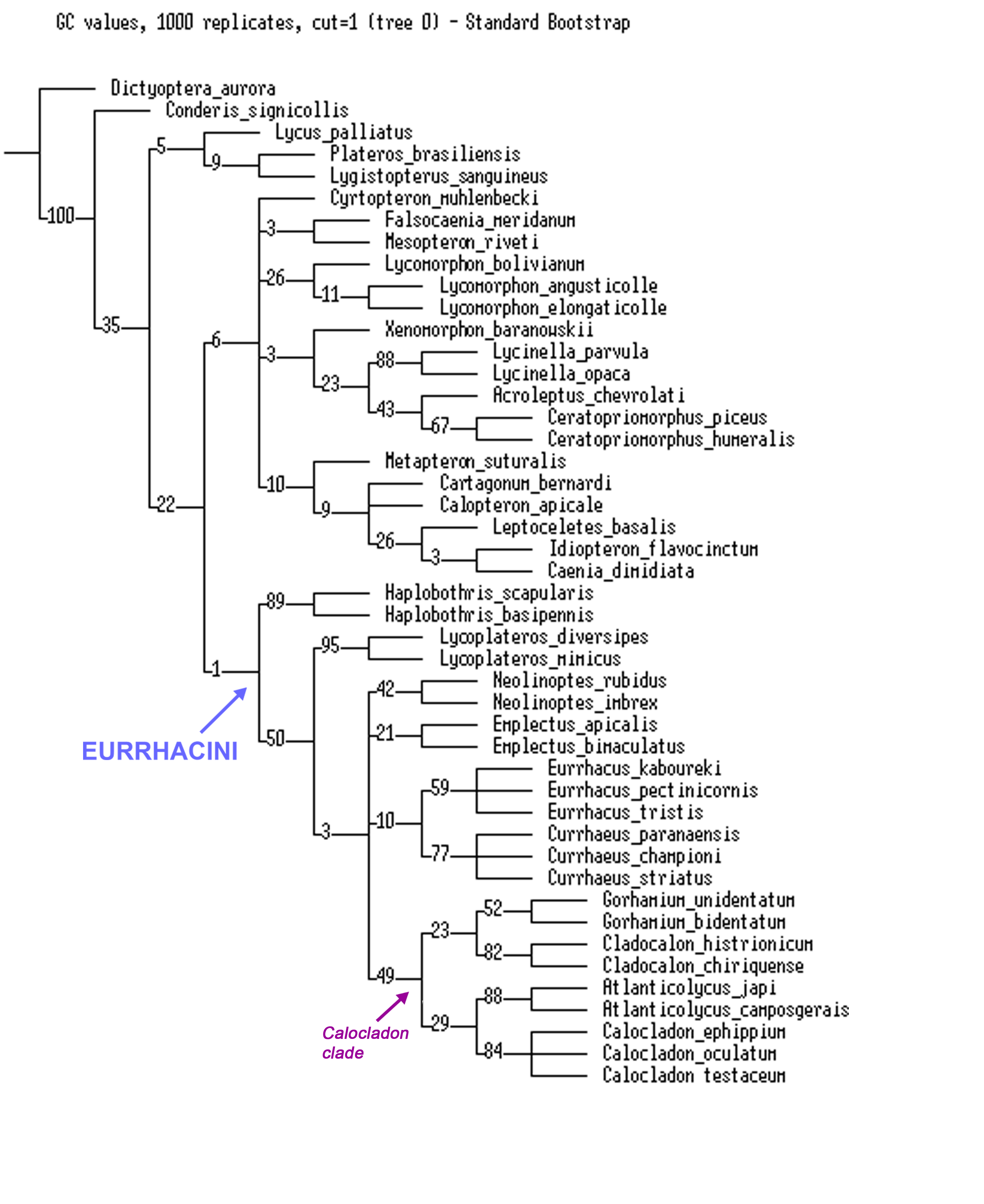

Supplement: Supplementary material 8 — Branch support using standard bootstrapping applied on the unweighted Calopterini-Eurrhacini dataset [file zookeys-1204-241_article-114932__-s008.tif]

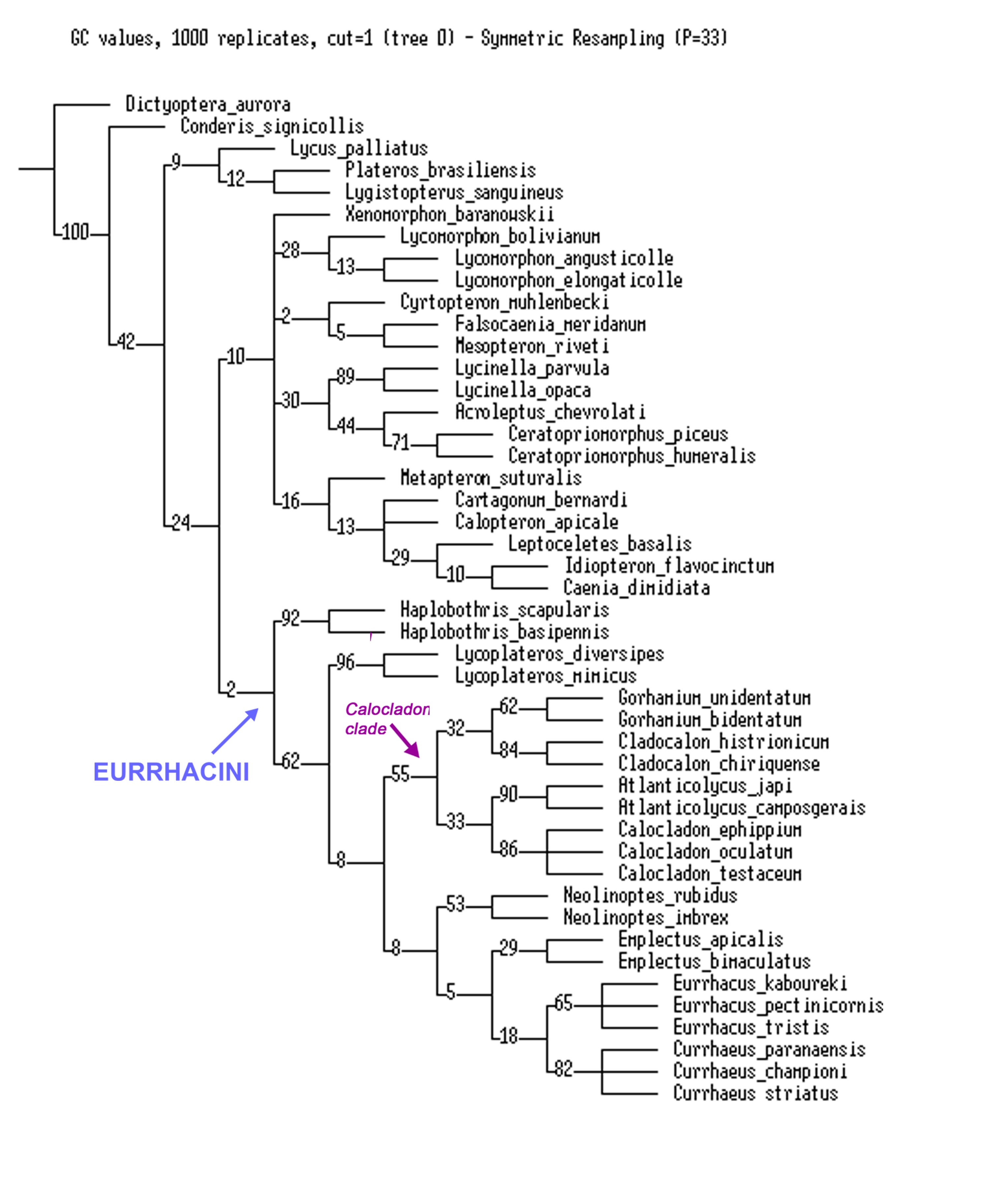

Supplement: Supplementary material 9 — Branch support using symmetric resampling applied on the unweighted Calopterini-Eurrhacini dataset [file zookeys-1204-241_article-114932__-s009.tif]

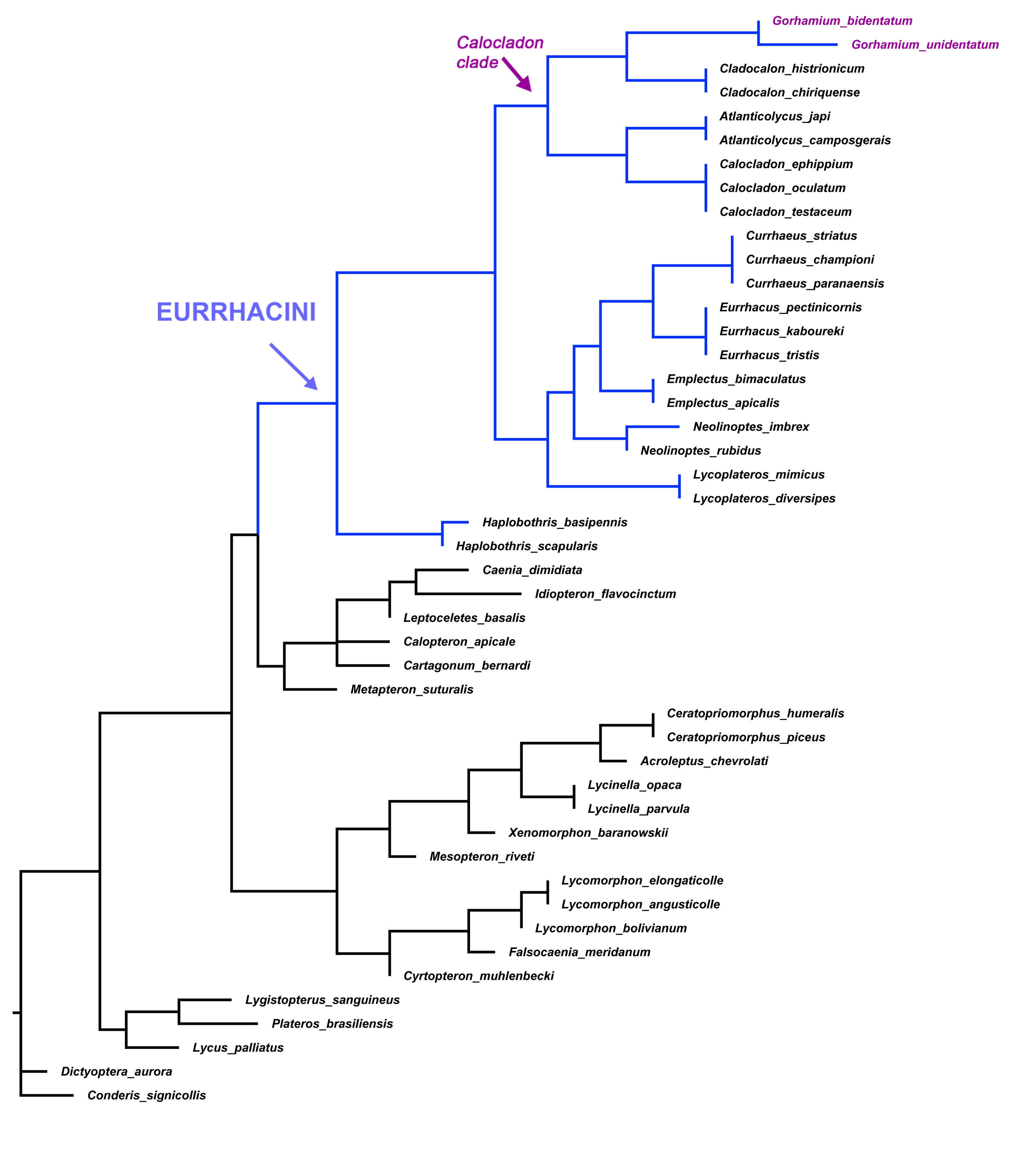

Supplement: Supplementary material 10 — The single implied weighted parsimony tree using TNT and the concavity constant in the range k = 3–25 [file zookeys-1204-241_article-114932__-s010.tif]
